# Electropolymerised PEDOT:Polydopamine enables high-performance bioelectrode coatings

**DOI:** 10.1038/s41598-025-19124-1

**Published:** 2025-10-08

**Authors:** Saloua Saghir, Jairo Ramirez-Sarabia, Kristin Imenes, Giuseppe Schiavone

**Affiliations:** Department of Microsystems, Faculty of Technology, Natural Sciences and Maritime Sciences, University of South-Eastern Norway, Horten, Norway

**Keywords:** Polydopamine, PEDOT, Conductive polymers, Electrode coatings, Adhesion, Electropolymerization, Biomedical engineering, Biomaterials, Sensors and biosensors

## Abstract

Conductive polymers are promising interface materials due to the ability to tailor conductivity, mechanics, and biocompatibility. A prevalent example is poly(3,4-ethylenedioxythiophene) doped with polystyrene sulfonate (PEDOT:PSS), a mixed-conduction composite widely employed as electrode coating. Despite widespread use, data indicate that PEDOT-based coatings could benefit from improved substrate adhesion. Here, we investigate polydopamine (PDA) as co-ion dopant for PEDOT, aiming at leveraging its adhesive properties. We develop a repeatable electropolymerization process for PEDOT:PDA on gold, and find performance metrics comparable to PEDOT:PSS, with large charge storage capacity of ~ 42 mC cm^−2^, effective interface capacitance of ~ 17.8 mF cm^−2^, and small variability. Sonication tests verify the adhesion advantage of PEDOT:PDA compared to PEDOT:PSS controls. We show manufacturability by integrating PEDOT:PDA in microfabricated devices, as well as scalability to electrode radii in the mm to 50 µm range. We anticipate the protocols and results herein to pave the way towards wider use of PDA in bioelectrode coatings.

**Supplementary Information:**

The online version contains supplementary material available at 10.1038/s41598-025-19124-1.

## Introduction

Conductive polymers (CPs) have emerged as versatile materials with applications spanning electrochemical biosensors, neuroelectronic interfaces, wearable bioelectronics, and tissue engineering scaffolds. Their appeal stems from a range of desirable properties, such as dual electronic-ionic electrical conductivity, tunable physicochemical properties, biocompatibility, and integration into micro- and nanofabrication processes^1,2^. Taken together, these capabilities render CPs ideal candidate materials for interfacing with biological systems and enabling advanced functionalities in bioelectronic medical devices. Among these materials, poly(3,4-ethylenedioxythiophene) (PEDOT) has been extensively studied since its first synthesis in the 1970s, as it displays large electrical conductivity when oxidized to its cationic form^3^. PEDOT can be synthesized by means of electropolymerization, a wet chemical process where an electrical potential is applied to induce the oxidation of the monomer, 3,4-ethylenedioxythiophene (EDOT), which polymerizes into PEDOT on an electrode surface^4^. This process requires an electrolyte to provide counterions, also known as co-ions, that balance the positive charge deposited on the PEDOT backbone during oxidation and are critical for preserving charge neutrality within the polymer matrix. A variety of PEDOT co-ions has been explored, including inorganic anions such as chloride^5^, or lithium^6^, organic anions such as p-toluenesulfonate (p-TS)^7^, and polymeric dopants such as poly(sodium 4-styrenesulfonate) (PSS)^4^, as well as biomolecules such as heparin, fibrinogen, or hyaluronic acid^8^.

A promising alternative co-ion for PEDOT is dopamine (DA), a catecholamine that plays a key role as neurotransmitter in the brain and messenger in peripheral tissue^9,10^. DA monomers can self-polymerize in aqueous media under alkali conditions, as well as electropolymerize under electrical potential. In its polymerized form, PDA demonstrates broad-spectrum surface adhesion, irrespective of substrate composition^11^; it is rich in functional groups such as catechol, carboxyl and amine groups, providing chemical reaction sites that can bind to biomolecules such as proteins and peptides to further promote affinity with biological systems^12,13^; and it is inherently biocompatible as it is found in the body.

Here, we test the hypothesis that PDA can be reliably used as co-ion of PEDOT in the synthesis of high-performance coatings for bioelectrodes. We posit that if successfully integrated with PEDOT in bioelectrode coatings, PDA can introduce the desirable advantages shown in its isolated form, namely adhesion, antibiofouling, intrinsic biocompatibility, and synthesis and deposition on electrode substrates by means of electropolymerization. First, we test different electrolyte chemistries and deposition parameters to develop a repeatable electropolymerization process for PEDOT:PDA coatings on gold electrodes. Next, we characterize coated electrodes in terms of electrochemical performance, topography, and adhesion, and show favorable comparisons with respect to PEDOT:PSS controls deposited following prior art protocols. Finally, we show the integration of PEDOT:PDA coatings into thin-film electrode arrays of the same class as typical flexible devices employed as neural interfaces in experimental studies, proving the scalability and manufacturability of the process developed herein.

## Results

### PEDOT:PDA electropolymerization

**Fig. 1:**
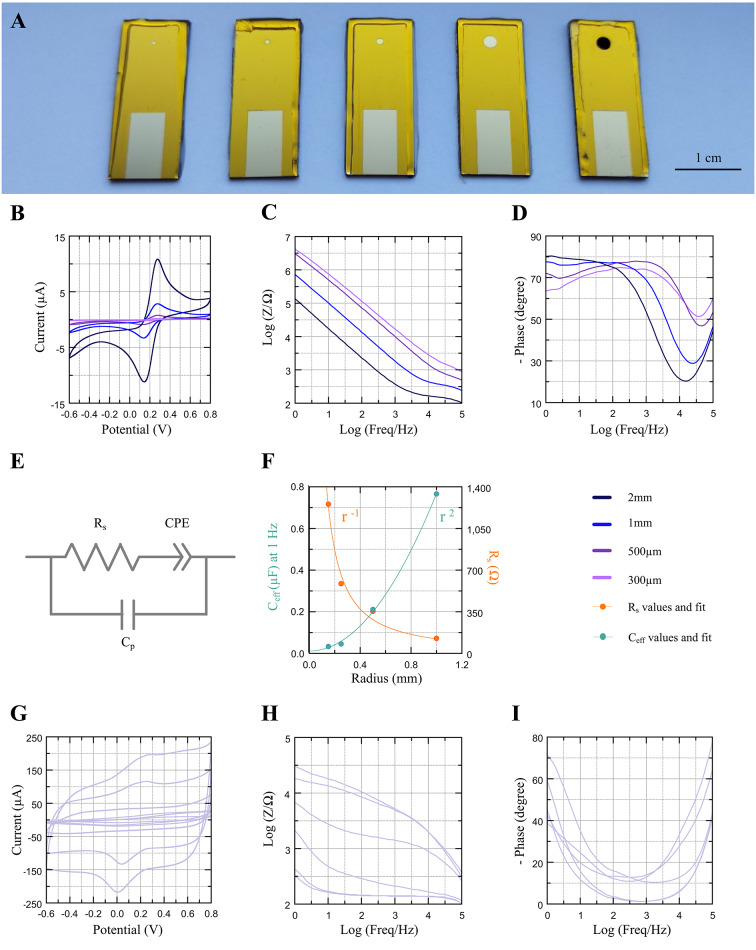
Electrochemical characterization of uncoated gold and PEDOT:PDA electrodes coated in pure PBS electrolyte. (**A**) Left to right, photographs of the 300 µm, 500 µm, 1 mm, 2 mm diameter bare Au test electrodes, and one 2 mm diameter PEDOT:PDA coated electrode. (**B**) CV scans of the bare Au electrodes at 100 mV s^-1^. (**C**) impedance modulus, and (**D**) impedance phase of the bare Au electrodes. (**E**) Equivalent circuit model used for EIS curve fitting. (**F**) Extracted parameter values of R_s_ and C_eff_ at 1 Hz for different electrode radii (dots). Trendlines: r^−1^ (R_s_) and r^2^ (C_eff_) (solid lines). Variability of performance in nominally identical PEDOT:PDA electrode samples (n = 6) coated in pure PBS electrolyte: (**G**) CV scans at 100 mV s^−1^; (**H**) Impedance modulus; and (**I**) Phase. All plots made with Labplot 2.10.0 (https://labplot.org/).

Round test electrodes were fabricated using thin-film Au sputter-deposited on thermally oxidized silicon wafers, insulated with Kapton tape, and accessed with via-openings patterned by means of CO_2_ laser ablation that define the electrode geometry (Fig. S1). We first verified that the Kapton tape provides the expected electrical insulation to the gold thin-film. To this end, via openings of different diameter 2r = 300 µm, 500 µm, 1 mm, 2 mm, were laser-cut in the Kapton insulation (Fig. 1A), and electrochemical measurements were taken on the resulting bare gold electrodes. Conventional electrochemical impedance spectroscopy (EIS) and cyclic voltammetry (CV) measurement techniques were used to characterize the electrodes, using standard setups and previously described conditions^14^. Here, we exploit the sensitivity of these measurements to electrode radius, to verify that our simple test electrode fabrication and insulation method enables sufficiently precise control on the electrode size. The impedance and CV trends for varying electrode diameters are shown in Fig. 1B–D. As expected, CV scans show a lower current response, while the EIS spectra display higher impedance for smaller diameter, compared to larger diameter electrodes. This was quantified by fitting the EIS curves to a simple lumped-parameter equivalent circuit model comprising of a series resistance R_s_ and a constant phase element (CPE) modelling the spreading resistance and the electrode–electrolyte interface, respectively (Fig. 1E).

We justify our simplified model with considerations on the physical nature of the test samples. Electrode interfaces can be modelled with a plethora of different equivalent circuits, the simplest of which includes a series resistor modelling the conductor and spreading resistances^14–16^, connected in series with a parallel circuit where one branch includes a charge transfer resistance modelling faradaic charge transduction, and the second branch includes a capacitor modelling capacitive charge transduction, typically using the Gouy-Chapman-Stern equation^17^. When analyzing the experimental spectra obtained within our chosen frequency range of 1 Hz – 100 kHz (Fig. 1C, D), we observe that capacitive effects dominate charge transduction at the interface, as shown by the slope of the low-frequency impedance modulus, and the corresponding impedance phase approaching 90°. This indicates a rather large charge transfer resistance that impedes current flow, while allowing a more favorable electrical path via the interface capacitance. Based on this consideration, we assume that the interface impedance can be modelled by capacitive effects only when f > 1 Hz. To further account for surface roughness and non-uniformity, we choose to replace the ideal interface capacitance with a constant-phase element of impedance

1 $${Z}_{CPE}=\frac{1}{{(j\omega )}^{n}Q},$$

where Q represents the magnitude of the quasi-capacitance, 0 < n < 1 is the exponent for the frequency dependency modelling surface inhomogeneities, and ω = 2πf is the angular frequency. To enable cross-sample and cross-study comparisons, we then use the fitting values for the CPE parameters Q and n to calculate an effective interface capacitance C_eff_ expressed in F. To this end, we choose a sample frequency and set the CPE impedance equal to the impedance of an ideal capacitor:

2 $${Z}_{CPE}=\frac{1}{j\omega {C}_{eff}}\Leftrightarrow {C}_{eff}=\frac{{\left(2\pi f\right)}^{n}}{2\pi f}\cdot Q=Q\cdot ({2\pi f)}^{\left(n-1\right)}.$$

As for the series resistance R_s_, contrary to the case of fully micropatterned devices, the conductor and connector resistances in our test samples are negligible compared to the electrode spreading resistance, which accounts for the volumetric resistance of the medium and electrode size. Therefore, we assume R_s_ in our equivalent circuit to model the spreading resistance only. Finally, we account for parasitic effects observed at high frequency, by adding a parasitic capacitance C_p_ in parallel to the series resistance and CPE. This addition models the rise of the impedance phase at high frequency due to parasitic capacitance effects in the test setup shunting the current away from the electrode–electrolyte path.

We use this simplified model (Fig. 1E) to extract fitting parameters and calculate the effective interface capacitance C_eff_ at 1 Hz, for different electrode radii r. The values for R_s_ and C_eff_ extracted from the measurement data are consistent with the theoretical trends of r ^−1^ and r ^2^, respectively^18^, as the spreading resistance follows inverse proportionality to the electrode radius, while the effective interface capacitance follows a direct proportionality with the electrode area, analogous to the case of a parallel plate capacitor. This is shown in Fig. 1F and confirms that the simple process of laser patterning the Kapton insulation enables sufficiently precise control on the electrode geometry.

Next, test devices with 2 mm diameter via openings were used as substrate for PEDOT:PDA electropolymerization. Electrodes were initially coated with PEDOT:PDA following previously reported EDOT and DA electropolymerization protocols in phosphate-buffered saline solution (PBS) at pH 7.2 by potentiostatic (PS) deposition^19^. Electropolymerization is a fast, cost-effective, and scalable technique for the deposition of conducting polymers, offering high spatial precision^20^ and conformability^21^, excellent control over film thickness^22^ and uniformity^23^, and substrate-specific patterning^24^, making it particularly well-suited for scalable microfabrication of bioelectronic interfaces^19^. This technique is commonly used for its simplicity^25^, as it allows the electropolymerization of the monomers in solution though the application of a constant potential between the substrate (working electrode) and a counter electrode. The resulting current flow and the corresponding charge are recorded as a function of time, so that the electropolymerization process can be stopped at a set charge, 50 mC in this case. Additionally, PS deposition has been reported to outperform other electrochemical deposition techniques for PEDOT, such as cyclic voltammetry^26^. Figure 1A shows one of the test electrodes coated with PEDOT:PDA using this protocol.

The first set of deposition tests confirms the ability to electropolymerize PEDOT:PDA on gold electrodes, however significant variability was observed when characterizing the electrochemical performance and coverage uniformity of the coatings on identical samples (n = 6 depositions in freshly prepared solutions). This is illustrated by the dispersion of the CV scans and impedance curves shown in Fig. 1G–I, with high variability in impedance modulus across the 6 samples (0.32–30.6 kΩ at 1 Hz, and 0.16–18.1 kΩ at 10 Hz). Optical micrographs of the coated samples in Fig. S2 additionally show unreliable surface coverage.

We hypothesized the inconsistency in these results to be due to the low solubility of EDOT in PBS (2.1 g L^−1^ in water at 20 °C)^27,28^. While ionic aqueous solvents (e.g. PBS, tris buffer, KCl)^21,29,30^ are extensively used to electropolymerize DA onto electrodes, several studies report low solubility of EDOT in aqueous media, making them unsuited as electrolytes for PEDOT electrodeposition^6,31^.

To address this challenge and identify a better suited electrolyte for PEDOT:PDA electropolymerization, we tested a range of alternative solvents that are known to dissolve both DA and EDOT^32–35^, while also being miscible with aqueous solutions. According to these criteria, we selected dimethyl sulfoxide (DMSO), N,N-Dimethylformamide (DMF), ethanol (EtOH), 2-propanol (IPA), methanol (MeOH), N-methyl-2-pyrrolidone (NMP), acetonitrile (ATN) and ethylene glycol (EG) as potential candidates, and we experimentally verify their ability to dissolve both DA and EDOT by observing solute residue in 1.5 ml solutions in Eppendorf vials. While solutions of EDOT in PBS typically exhibit undissolved droplets or floating residue (Fig. S3A), all the chosen solvents could effectively dissolve both EDOT and DA, with the exception of ATN, in which DA was not dissolved. Nevertheless, ATN was not excluded from electropolymerization experiments, as previous literature report it to affect the morphology of PEDOT coatings^34,36^, making it a variable of interest (SEM images available in Figure S4). Additionally, by introducing solvents at lower pH, no DA self-polymerization was observed even after two weeks, as opposed to PDA self-polymerizing within 24 h in PBS at pH 7.2. Photographs of DA dissolution in different solvents and corresponding pH values are displayed in Fig. S3B and Table S1, respectively.

**Fig. 2:**
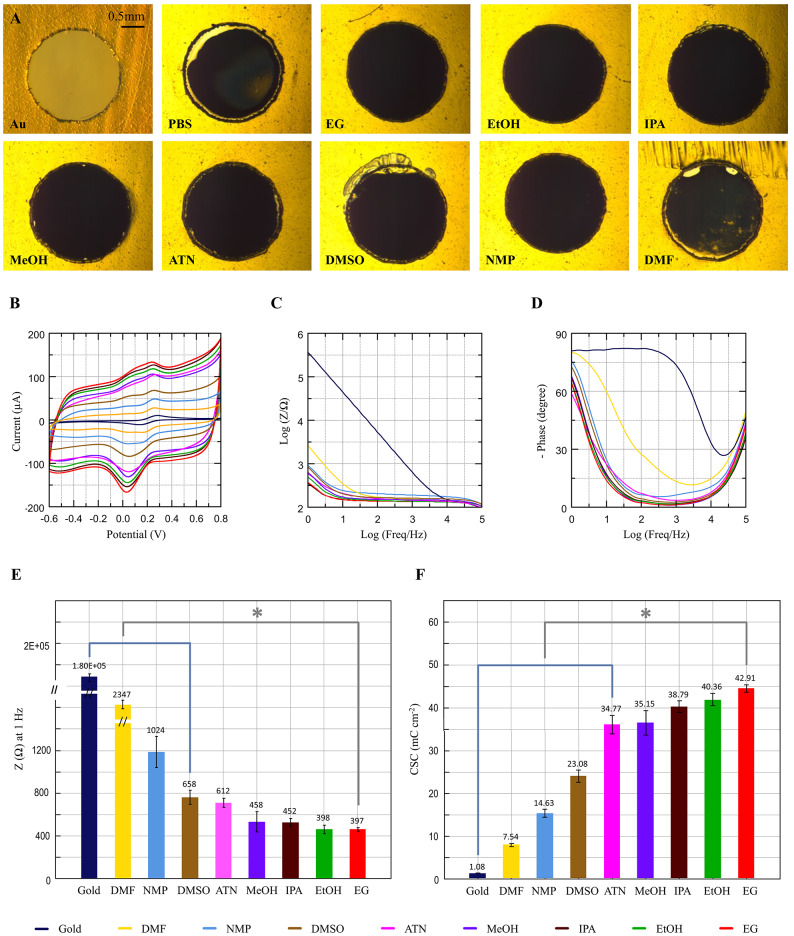
Characterization of the PEDOT:PDA electrodes electropolymerized in different electrolyte solvents. (**A**) Representative optical micrographs of 2 mm diameter PEDOT:PDA electrodes coated using different electrolyte solvents. (**B**) CV scans in [Fe(CN)_6_]^3−/4−^ at 100 mV s^−1^ scan rate. (**C**) Impedance modulus and (**D**) phase for the different solvents. The curves shown are averages over n = 4 individual samples. (**E**) Bar plot of the impedance modulus at 1 Hz. Bar height: mean. Error bars: standard error. (**F**) Bar plot of the CSC values. Bar height: mean. Error bars: standard error. * *p *< 0.05, Mann–Whitney U test.

2 mm diameter gold electrodes were coated by potentiostatic deposition in electrolytes containing the different solvents above. We initially identified suitable electrolyte compositions by running deposition tests aimed at selecting adequate monomer concentrations, PBS:solvent volumetric ratios, and deposition charge. We then evaluated the coatings according to deposition coverage and electrochemical characterization. The results, shown in Figs. S5 and S6, indicate an optimal electrolyte composition comprising of 1 mg mL^−1^ DA and 10 mM EDOT monomer concentrations, 90:10%v:v PBS:solvent ratio, and a deposition charge of 50 mC. Next, a total of n = 4 electrode samples were coated for each electrolyte solvent, using the electrolyte compositions identified above. Optical microscopy photographs of the coated samples for the different solvents are shown in Fig. 2A. A typical black coloration of the gold electrode was observed indicating the presence of the PEDOT:PDA film. The electrodes coated in pure PBS and NMP display less uniform coatings with regions of uncoated gold, while uniform PEDOT:PDA coverage was observed for samples electropolymerized using the other solvents. All coated electrodes were then characterized by CV and EIS (Fig. 2B, C, D). The EIS spectra taken on coated electrodes display quasi-capacitive behavior in the low frequency range (< 10 Hz), and a dominating resistive behavior at frequencies larger than 10 Hz. When compared to bare gold electrodes, where the smaller interface capacitance shifts the sloped EIS capacitive impedance region upwards and visible even at high frequencies <  ~ 10 kHz, these results show that the PEDOT:PDA coatings achieve considerable drops in impedance modulus in the range of 99.36% (2347 Ω) –99.89% (397 Ω) at 1 Hz (compared to 180 kΩ at 1 Hz for bare Au), depending on the electrolyte composition. The electrochemistry characterization data further confirm consistency between CV current responses and impedance spectra, with lower current samples as identified in the CV plots exhibiting a corresponding higher impedance modulus in the low-frequency range, where both measurements are governed by similar charge transduction mechanisms^37^. Except for DMF, DMSO and NMP samples, the 1 Hz impedance modulus variations across samples electropolymerized with all other solvents were comparable to intra-group variability, as displayed by the error bars in Fig. 2E. This is quantified by Mann–Whitney U tests (p-values reported in Tables S2 and S3), which reveal that the results for MeOH, EtOH, IPA, and EG cannot be significantly differentiated (*p* > 0.05), as opposed to lower-performing solvents such as DMF, DMSO, and NMP, for which statistically significant differences were detected (*p* < 0.05).

### Electrochemical performance metrics

Next, we used the electrochemistry measurement data to extract performance metrics, with the aim of quantifying differences between the coatings and identifying criteria to select electropolymerization processes that yield the best results. We used the CV data to calculate the cathodic charge storage capacity (CSC) of each sample, a metric that quantifies the total negative electric charge the electrode can store and release over a complete voltage cycle. This is a typical benchmark of the electrode performance for capacitive energy storage^38^ or neural recordings^37^, with larger CSC indicating that the electrode can store more charge compared to smaller CSC electrodes. CSC values calculated for the coated electrodes are listed in Table 1. All the coated samples display significantly larger CSC compared to the uncoated bare Au controls (1.08 ± 0.02 mC cm^−2^), with values ~ 7 × to ~ 40 × in the worst and best cases. PEDOT:PDA coatings electropolymerized using the ethylene glycol electrolyte exhibit the largest CSC (~ 43 mC cm^−2^ mean), followed by the Ethanol, IPA and Methanol samples. Notably, the variation in CSC among these solvents was comparable to the corresponding intra-group variability.

**Table 1:** CSC and EASA values for PEDOT:PDA electropolymerized using different solvents. Values are expressed as mean ± standard error.

Solvent	ATN	DMF	DMSO	EG	EtOH	IPA	MeOH	NMP
CSC [mC cm^−2^]	34.77 ± 4.18	7.54 ± 0.80	23.08 ± 2.74	42.91 ± 1.74	40.36 ± 2.83	38.79 ± 2.58	35.15 ± 5.51	14.63 ± 1.77
EASA [cm^2^]	0.23 ± 0.01	0.06 ± 0.01	0.15 ± 0.02	0.29 ± 0.02	0.25 ± 0.02	0.26 ± 0.01	0.23 ± 0.03	0.10 ± 0.01
EASA/GSA	~ 7	~ 2	~ 5	~ 9	~ 8	~ 8	~ 7	~ 3

For comparison, we coated 3 test electrodes with PEDOT:PSS using a 1:2.5 EDOT:PSS ratio, as previously reported^25^, and EDOT concentration maintained at 10 mM. The deposition was stopped when the charge density reached 200 mC cm^−2^, based on values reported in previous studies^39,40^. Optical microscopy photographs and CV plots taken on the PEDOT:PSS control samples are shown in Fig. S7. The resulting CSC values were extracted as 12.99 ± 0.26 mC cm^−2^, falling within the typical range of 10 mC cm^−2^ ^26,41^ to 100 mC cm^−2^ ^25,42^ reported in the literature. Compared to our own PEDOT:PSS controls, the best PEDOT:PDA coatings shown here achieve ~ 3 × larger CSC values.

To complement the CSC results, we used the [Fe(CN)_6_]^3−/4−^ CV data to calculate the electrochemically active surface area (EASA) of all the samples, using the Randles-Ševčík equation (see methods). The calculated values are shown in Table 1 and confirm trends consistent with what observed for the CSC values. We quantify the improvement in EASA introduced by the PEDOT:PDA coatings compared to the geometrical surface area (GSA, ~ 0.031cm^2^). The results in Table 1 show EASA/GSA ratio improvement in the range of 2–9.

**Fig. 3:**
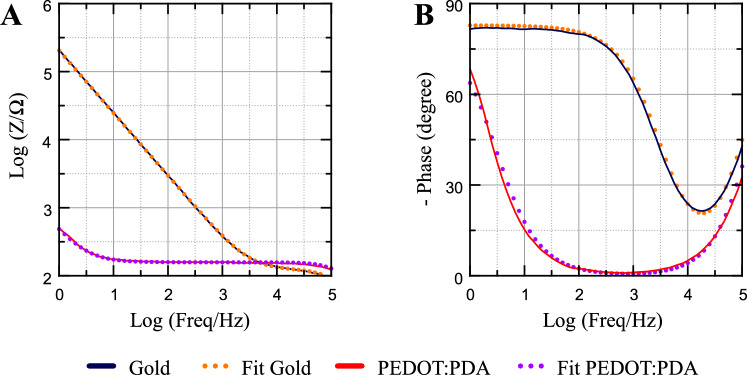
EIS curve fitting for Au and EG-electropolymerized PEDOT:PDA electrodes. **(A)** Impedance modulus and **(B)** phase curves of Au and EG-electropolymerized PEDOT:PDA electrodes. Solid lines: measurements. Dotted lines: model fits.

To further quantify the improvement introduced by PEDOT:PDA coatings, we fit the EIS data to the equivalent circuit model in Fig. 1E and extract the parameter values for bare gold and EG-deposited PEDOT:PDA, chosen as best-performing based on the CSC and EASA data (Fig. 3). The fitting parameters (Table S6) confirm that, as expected, the coating i) does not considerably affect the series resistance R_s_ of the system, as this is largely dominated by the GSA-governed spreading resistance^18^, which is equal in both sample groups; while ii) significantly increasing the effective interface capacitance C_eff_ of the electrode at 1 Hz by almost 3 orders of magnitude, from ~ 0.7 µF for Au to ~ 0.36 mF for PEDOT:PDA. Our proposed equivalent circuit model is in line with previously reported approaches used to describe the electrochemical behavior of PEDOT-based coatings^4,19^ where similar configurations were employed to capture interfacial charge transfer and capacitive behavior.

Based on this set of results, 4 electrolyte solvents (EtOH, IPA, EG and MeOH), were identified as enabling electrode coatings with the most enhanced current response and reduced impedance. Within these sample groups, the differences observed in the electrochemical metrics were negligeable, especially when compared with typical performance drops observed in real-world biological applications. In implanted electrodes, for instance, it is expected that tissue encapsulation growth over time would represent a dominant factor affecting CSC and EIS measured in vivo (up to 5–10 × change from pristine)^14,43,44^. Based on this, we narrow the chemistry selection to the 4 electrolyte solvents above to conduct further characterization.

### Topography characterization

**Fig. 4:**
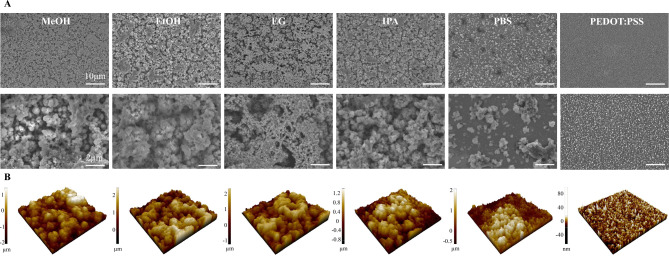
SEM images of PEDOT:PDA coated electrodes. (**A**) Representative SEM images of PEDOT:PDA electropolymerized using different solvents, and PEDOT:PSS control. Scale bars: 10 µm (top row) and 2 µm (bottom row). (**B**) Corresponding 3D surface reconstructions from AFM scans (25 µm^2^ scan area).

PEDOT:PDA electrodes coated using the four best-performing electrolyte solvents were imaged by SEM and AFM to characterize surface topography, and quantify surface roughness, respectively. These complementary imaging techniques were also used to assess the uniformity of the coating and verify that the electrode surface was entirely covered. SEM scans of representative samples are shown in Fig. 4A, displaying a rough and porous surface that explains the results on EASA increase compared to bare Au. No qualitative difference was observed in the surface topography across samples electropolymerized with the 4 different solvents. However, the surface roughness appears larger compared to samples coated using PBS-only electrolytes, which display a smoother surface with patches of rough polymerized PEDOT:PDA distributed across the electrode. In contrast, the solvent-assisted coatings display a highly porous matrix with multiscale pore distribution and entangled structural network, as shown in high magnification SEM images (Fig. 4A).

To corroborate the SEM observations, the samples were next characterized by AFM, with the resulting 3D images showing large surface roughness with micrometer-scale dip-to-peaks for all solvent-mediated depositions (Fig. 4B). In contrast, samples coated in pure PBS present flatter regions, as indicated by larger dark red areas on the AFM image, consistent with the SEM characterization. Small differences in surface roughness were measured across samples deposited using different solvents, as shown by the arithmetic average roughness R_a_ values in Table 2. PEDOT:PSS control samples displayed a smoother surface compared to PEDOT:PDA, as shown both in the SEM and AFM images, with nanometer-sized grains and less porous surface structures.

**Table 2:** Thickness and surface roughness measured on PEDOT:PDA samples electropolymerized using the four selected solvents, pure PBS electrolyte, and PEDOT:PSS control.

	MeOH	EtOH	IPA	EG	PBS	PEDOT:PSS
Thickness [nm]	2176.66 ± 72.93	4311.32 ± 71.00	3419.51 ± 35.96	4232.96 ± 10.27	1657.03 ± 944.42	755.99 ± 24.75
R_a_ [nm]	423	443	441	495	443	6.61

Thickness measurements were additionally taken for each sample by means of mechanical profilometry, with results ranging from 2176.66 ± 42.11 nm to 4232.96 ± 10.27 nm. The variability on the thickness measurements is larger than the variability observed on both the surface characterization and electrochemical metrics, suggesting that electrode performance is dominated by surface, rather than bulk processes. This confirms the validity of the chosen electrochemical performance metric of C_eff_, which is typically representative of surface capacitive processes.

### Coating adhesion

**Fig. 5:**
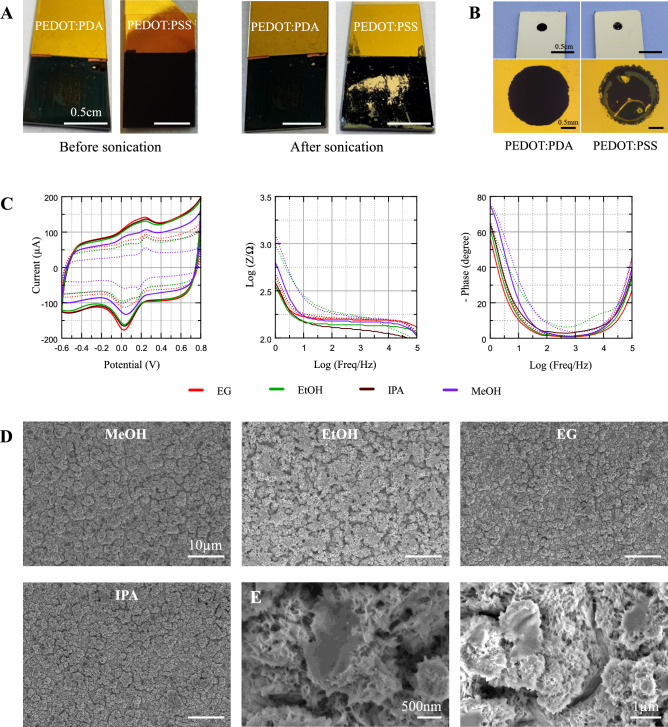
Optical and electrochemical characterization of coated electrodes after sonication. (**A**) Photographs of 1 cm^2^ PEDOT:PDA and PEDOT:PSS coated electrodes before and after ultrasound treatment. (**B**) Photographs of coated electrodes after stripping the Kapton tape encapsulation (top) and optical microscopy images of the electrodes after ultrasound treatment. (**C**) (Left to right) CV scans at 100 mV s^−1^; impedance modulus; and impedance phase plots before (solid line) and after (dashed line) sonication. (**D**) Representative SEM images after sonication (scale bars: 10 µm). (**E**) SEM images of de-structured patches observed after sonication.

Once the electrochemical and surface properties of the PEDOT:PDA coatings were confirmed, we tested the hypothesis that replacing PSS with PDA as a co-ion of PEDOT enables the electrochemical performance of PEDOT to be combined with the adhesion properties of PDA. First, tape tests were performed on 1 cm^2^ square samples of PEDOT:PSS and PEDOT:PDA coated on sputtered gold electrodes, using both regular Sellotape and Kapton tape covering the entire electrode surface. No delamination nor residue on the tape was optically observed on either type of coating, indicating that the electropolymerization process provides good adhesion on as-fabricated, pristine electrodes. Next, 1 cm^2^ square test electrodes were immersed in a vessel containing deionized water heated at 35 °C and exposed to 37 kHz ultrasound sonication (320 W peak power) for 20 min, following previously reported test methods^45^. At the end of the test, the PEDOT:PSS coating showed delamination from the Au substrate, whereas PEDOT:PDA remained intact, even when subjected to sonication for 10 additional minutes (Fig. 5A). We then proceeded to test adhesion on coatings electropolymerized on smaller electrodes, patterned by via-opening as described earlier. To this end, we coated 2 mm diameter electrodes with PEDOT:PDA using the 4 selected electrolyte solvents, as well as PEDOT:PSS controls. All samples (n = 2 per type) were immersed in the heated sonication bath for 1 h, and no delamination was observed on any sample. Next, the 65 µm-thick Kapton tape encapsulation was stripped to avoid the via sidewall acting as protection for the coating, to mimic real-life applications of devices with conductors protected with few-µm thick insulation layers. Samples with the insulation stripped were then exposed to the same heated sonication bath. After 10 min, delamination was observed on the PEDOT:PSS, but not on the PEDOT:PDA samples (Fig. 5B), even after prolonging the sonication treatment for 10 additional minutes.

CV and EIS curves were taken on pristine samples, and before stripping the encapsulation. The data is shown in Fig. 5C and shows CSC decrease post-/pre- sonication of around 28, 21, 29 and 38% for EG, EtOH, IPA, MeOH samples, respectively. Similarly, 1 Hz impedance moduli are shown to increase post-/pre- sonication by 22, 86, 22, 28% for EG, EtOH, IPA, MeOH samples, respectively.

To visualize the effect of the sonication on the PEDOT:PDA coating topography, the samples were further imaged by SEM, revealing that the rough and porous surface observed on pristine samples was maintained overall (Fig. 5D). Only minor structural changes were identified, with some regions appearing to collapse into micrometer-sized aggregates and micrometer-sized patches of smoother PEDOT:PDA scattered across the surface appeared (Fig. 5E). This phenomenon was more pronounced in samples that exhibited a greater decrease in electrochemical performance.

We ascribe the enhanced adhesion observed for PEDOT:PDA in comparison to PEDOT:PSS to the presence of catechol and primary amine groups introduced by PDA^46,47^. Catecholamines have the ability to attach to metal surfaces via coordination bonds and hydrogen bonds^48–50^, as confirmed in other studies where incorporating PDA into conducting polymers has been reported to enhance interfacial adhesion on metal and metal-oxide surfaces^51–53^. Taken together, these results confirm the hypothesis that using PDA as co-ion of PEDOT introduces an adhesion advantage compared to standard PEDOT:PSS controls, with potential positive implications to future device reliability.

### Electrode polarization under current pulsing

**Fig. 6:**
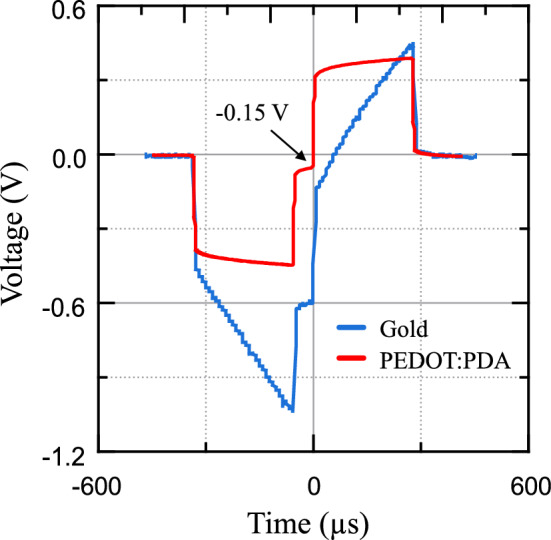
Voltage transient curves for gold and EG-deposited PEDOT:PDA electrodes at 1.5 mA and 10 mA current amplitude, respectively.

Numerous bioelectrode applications revolve around electrical stimulation of biological tissue by means of charge injection through current pulsing. The performance of electrodes when subjected to current-controlled pulsing is only loosely correlated with the CSC and impedance metrics quantified in previous section^14,37^, as the charge transduction regimes are different between the cases of slow-rise voltage excitation and square-pulse current injection. Therefore, in order to assess the electrical stimulation performance of PEDOT:PDA coatings, we conducted voltage transient measurements to record the interface polarization of test electrodes when subjected to current-controlled pulsing. Cathodic charge injection capacity (CIC) is a metric that complements CSC in describing the performance of an electrode, as it quantifies the maximum amount of charge per unit area that an electrode can deliver through a specific stimulation waveform, without exceeding a predetermined interface polarization threshold. For bioelectrodes, the interface polarization threshold is typically chosen as the cathodic water window limit, corresponding to a voltage of − 0.6 V against a Ag|AgCl reference electrode. We used charge-balanced, biphasic, symmetrical, cathodic-leading, current-controlled pulses with 0.3 ms pulse width and 60 µs inter-phase delay to generate a singularity point in the polarization time-plot^14^. When measuring voltage transients on 2 mm diameter EG-deposited PEDOT:PDA samples, the maximum available current amplitude of the pulse stimulator was delivered (−10 mA, corresponding to a charge density of ~ 95 µC cm^−2^), with the interface polarization reaching approximately −0.15 V (Fig. 6). In comparison, an uncoated bare Au electrode reached the cathodic water window limit of −0.6 V with 1.5 mA amplitude pulses (corresponding to a CIC limit of ~ 14 µC cm^−2^). The low interface polarization achieved while delivering large current amplitude pulses contributes to i) widening the current range for electrical stimulation before reaching the electrochemical safety limit of water electrolysis at the interface; and ii) lowering the voltage compliance requirements of stimulation electronics, with specific reference to battery-powered implanted pulse generators. The low interface polarization value measured for PEDOT:PDA suggests a CIC limit much larger than 100 µC cm^−2^, which although could not be precisely measured due to hardware limitations on the current output of the pulse stimulator, confirms the potential of PEDOT:PDA as promising interface material for voltage-efficient electrical stimulation.

### Manufacturability

#### Repeatability

**Fig. 7:**
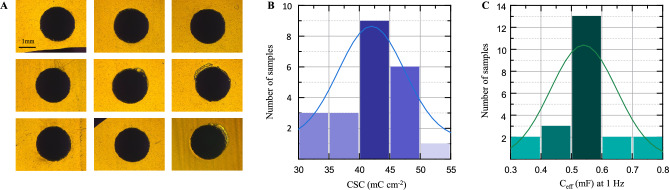
Repeatability of the PEDOT:PDA electropolymerization process. (**A**) Representative optical microscopy images of nominally identical PEDOT:PDA coated electrodes. Distribution plots of the extracted values of (**B**) CSC, and (**C**) C_eff_ at 1 Hz.

One of the challenges initially addressed in this study was the large batch-to-batch variability observed when electropolymerizing PEDOT:PDA in pure PBS, both in terms of coating uniformity and electrochemical performance. Once improved electropolymerization protocols were developed and validated, we chose one of the electrolytes yielding best-performing electrode coatings (90:10%v:v PBS:EG) and tested its repeatability by depositing a total of n = 22 nominally identical samples. Uniformity of the coating was confirmed on all samples as shown in the optical microscope images of representative samples in Fig. 7A. We confirmed the repeatability of the process by quantifying the distribution and variability of performance metrics measured across the whole sample group. CSC and C_eff_ at 1 Hz parameter values extracted from CV and EIS data were tested for normality (Shapiro–Wilk test, W = 0.937, *p* = 0.172 ≫ 0.05, and W = 0.972, *p* = 0.772 ≫ 0.05, for CSC and Ceff, respectively), and Fig. 7B, C shows the corresponding bar plot distributions. The CSC and C_eff_ at 1 Hz values extracted for all samples are listed in Table S7, amounting to mean and standard error of 42.00 ± 0.16 mC cm^−2^ and 0.560 ± 0.021 mF, respectively.

#### Scalability

**Fig. 8:**
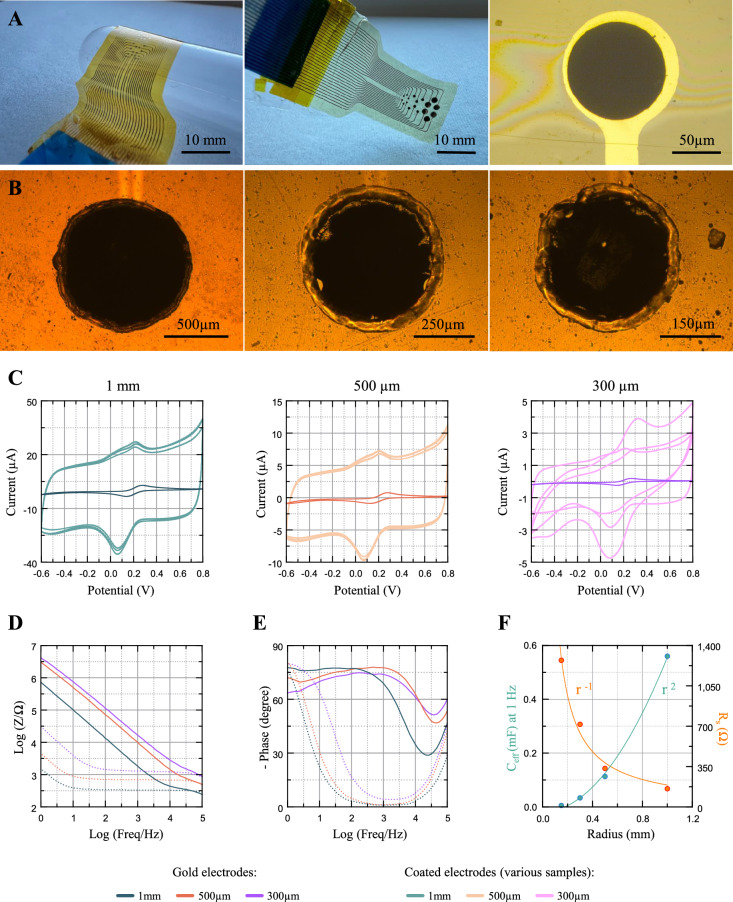
Scalability of PEDOT:PDA electropolymerization technology across electrode diameters. (**A**) Left to right: photographs of a flexible multi-size electrode array device before and after PEDOT:PDA coating, and optical micrograph of a 100 µm diameter coated electrode. (**B**) Left to right: optical micrographs of 1 mm, 500 µm and 300 µm diameter test electrodes. (**C**) Left to right: CV scans (100 mV s^−1^) of 1 mm, 500 µm, and 300 µm diameter coated electrodes. (**D**) Impedance modulus and (**E**) phase plots for 1 mm, 500 µm and 300 µm gold (solid lines) and coated (dashed lines) electrodes. (**F**) EIS fitting parameter values of R_s_ and C_eff_ at 1 Hz for different PEDOT:PDA electrode radii. Trendlines: r ^−1^ (R_s_) and r ^2^ (C_eff_ at 1 Hz).

Different applications require a variety of electrode sizes, ranging from cm-scale human ECoG grids or spinal stimulation paddles, to few µm diameter electrodes for high-resolution neural recordings or high-sensitivity electrochemical sensing. To accommodate such a broad application spectrum, material technologies should be accompanied by a corresponding scalability of the associated manufacturing process. We demonstrate the integration of our PEDOT:PDA electropolymerization process by fabricating a flexible electrode array device using a standard thin-film gold on polyimide microfabrication process^54–56^, with different electrode diameters defined by photolithography. Figure 8A shows the successful fabrication of one such device integrating PEDOT:PDA coated electrodes of 1 mm, 500 µm, 300 µm, 100 µm and 50 µm diameters.

To assess the change in electrode performance in controlled test conditions, we separately tested electropolymerization on electrode diameters of 2 mm, 1 mm, 500 µm and 300 µm, covering the midrange of the aforementioned dimensional spectrum. To enable direct comparison, the charge density in each PS deposition was maintained constant across electrode sizes, and equal to the value chosen for the previous experiments (50 mC for 2 mm diameter electrodes, corresponding to ~ 1.58 C cm^−2^). The deposition charge was then calculated accordingly, and n = 3 test electrodes were fabricated for each electrode size (representative micrographs in Fig. 8B).

Compared to bare Au counterparts, 1 mm and 500 µm diameter PEDOT:PDA electrodes display increases of ~ 35- and ~ 40-fold in CSC, and seven and eightfold in EASA, respectively, as calculated from the CV curves of Fig. 8C. The data is listed in Table 3, showing remarkably small variability. 300 µm diameter electrodes showed higher intra-group variation due, we infer, to capillary effects around the electrode perimeter caused by the thickness of the Kapton tape (65 µm). This phenomenon is avoided when the electropolymerization deposition is integrated into thin-film microfabrication and lithography processes such as the one used for the fabrication of the flexible electrode array in Fig. 8A, where via depth is typically limited to few µm’s. Despite this challenge, the GSA-normalized performance metrics of CSC and EASA/GSA listed in Table 3 show remarkable consistency across the different electrode size, and the C_eff_ at 1 Hz values extracted from the EIS curves (Fig. 8D, E) closely follow the expected r^2^ trend (Fig. 8F). Taken together, these results concertedly confirm the scalability of our PEDOT:PDA material technology and its broad applicability to various dimensional designs.

**Table 3:** Scalability of CSC and EASA/GSA metrics across electrode diameter.

Electrode diameter	2 mm	1 mm	500 µm	300 µm
CSC [mC cm^−2^]	42.00 ± 1.16	34.92 ± 0.78	39.60 ± 0.63	29.70 ± 8.57
EASA [cm^2^]	0.29 ± 0.02	0.055 ± 0.002	0.015 ± 0.001	0.0056 ± 0.0013
EASA/GSA	~ 9	~ 7	~ 8	~ 8

## Discussion

This study investigates the use of polydopamine as co-ion for PEDOT-based bioelectrode coatings, quantifies their performance and identifies advantages and challenges compared to state-of-the-art PEDOT:PSS technology. The rationale behind choosing PDA as PEDOT dopant stems from its renown adhesive properties, antibiofouling effects, and compatibility with deposition by electropolymerization. Starting anew from a range of possible electrolyte chemistries, we developed a repeatable process for the potentiostatic deposition of PEDOT:PDA coatings on gold electrodes. We optimized the process against a range of different electrolyte solvents to dissolve both EDOT and DA, their volumetric ratio with respect to PBS buffer, concentration of EDOT and DA monomer, and total deposition charge. Based on our results, we select ethylene glycol as solvent, and monomer concentrations of 10 mM EDOT and 1 mg mL^−1^ DA. The details of our optimized electrolyte composition and process parameters are listed in Table S8 and will serve as an easily transferrable protocol for other research groups to utilize our material technology in further applications.

Following our process, we achieved high-performance electrode coatings showing significant improvement compared to uncoated controls, with large charge storage capacity of ~ 42 mC cm^−2^ and effective 1 Hz interface capacitance of ~ 0.56 mF on average, which compare favorably to other modern high-performance material technologies such as PEDOT:PSS^26,57^, IrOx^58,59^, or roughened Pt^60^. Topography characterization by SEM and AFM revealed a rough surface morphology that supports the measured increase in interface capacitance, and the consequent reduction in the low-frequency impedance. Such rough morphology was intentionally promoted through the electropolymerization conditions, as it enhances the electroactive surface area and contributes to the improved electrochemical performance of the coatings.

We confirmed the adhesion advantage provided by PDA when electropolymerized with PEDOT through sonication tests, where standard PEDOT:PSS controls exhibited delamination while PEDOT:PDA samples remained intact, with slight decrease in their electrochemical performance metrics. We further validated the manufacturability of our material technology by showing repeatable depositions with small variability between namely identical samples, and scalability across the mm to 50 µm electrode radii, as well as integration of the electropolymerization step into conventional microfabrication, whereby we achieved fabrication of flexible, multisize PEDOT:PDA coated electrode arrays using a standard thin-film metal-on-polyimide photolithography process.

Taken together, our results constitute a solid base for future refinement and field-validation of PEDOT:PDA technology in a wide range of applications, spanning electrochemical sensors that exploit the functionalization capabilities of PDA^61,62^, and bioelectronic interfaces that leverage the high electrochemical performance and low interface polarization of the coating. Further research will aim at confirming other desirable properties of PDA when used as co-ion of PEDOT in electrode coatings, including testing whether PEDOT:PDA can retain the antibiofouling properties seen in isolated PDA, as well as ensure (bio)stability under different use conditions. A simple preliminary immersion test in PBS at 57 °C shows promising results (Fig. S8).

Previous research has already explored the incorporation of PDA into conductive polymers as a method to improve performance in different applications, such as stability of perovskite for solar cells^63^, biosensor selectivity^64,65^, textile sensor durability^66^, neuromodulation^19,51^, wettability in supercapacitors^67^, coating adhesion in flexible electronic devices^52^, cell attachment and expansion^68^. The repeatability, reliability and consistent high-performance metrics that we report in this work will play a fundamental role in enabling broader application of PEDOT:PDA bioelectrode coatings by a larger user base. From a more general standpoint, this work proves viability and underscores the potential of integrating biomaterials such as PDA, into scalable fabrication processes that are typically associated with the realm of MEMS and Si manufacturing.

## Materials and methods

### Chemicals

N,N-Dimethylformamide (DMF) (319937), dimethyl sulfoxide (DMSO) (D8418), N-Methyl-2-pyrrolidone (NMP) (M79603), ethylene glycol (EG) (293237), 3,4-ethylenedioxythiophene (EDOT) (483028), acetonitrile (ATN) (360457), dopamine hydrochloride (H8502), potassium hexacyanoferrate (III) (K_3_Fe(CN)_6_) (244023), potassium chloride (KCl) (P9541), poly(sodium 4-styrenesulfonate) sodium salt (PSS) (243051) were purchased from Sigma Aldrich. 2-propanol (IPA) (109634), ethanol absolute (EtOH) (20821), and methanol (MeOH) (113351) were sourced from VWR, Norway. Gibco phosphate buffered saline × 1—pH7.2 (PBS) (20012027) was acquired from Thermo Fisher Scientific.

### Fabrication of the electrodes

Silicon wafers were thermally oxidized to grow a 200 nm layer of silicon dioxide (Harmbridge Hitech Furnace). Subsequently, a 10 nm layer of chromium was deposited at a rate of 0.1 Å/s to serve as an adhesion layer, followed by the deposition of 100 nm of gold at a rate of 0.65 Å/s by magnetron sputtering (Sputter AJA ATC). Kapton (RND Electronics) tape was laminated onto the wafers and then laser-patterned (Zing 24, Epilog Laser). The Kapton tape was selectively removed to expose the working electrodes and contact pads. The diameters of the electrodes range from 2 mm, 1 mm, 500 µm to 300 µm. To eliminate Kapton adhesive residues, the wafers were immersed in isopropanol and subjected to ultrasonic cleaning for 3 min. The wafers were then diced into individual chips (Disco DAD 3220), and the edges of the chips were passivated with silicone (Dow Corning 3140 RTV) to avoid delamination of the Kapton tape from the corners and any residual electrically conductive areas.

### PEDOT:PDA electropolymerization

The electropolymerization of PEDOT:PDA was performed in a three-electrode cell with a silver/silver chloride reference electrode, a 2 cm^2^ platinum mesh counter electrode, and various working electrodes under continuous magnetic stirring, using a CH Instruments (CHI660E) potentiostat. Volumetric solvent ratios are listed in table S9. PBS-only electrolyte: Dopamine hydrochloride (1 mg mL^−1^) and EDOT (10 mM) (10.7 µL total) were dissolved in 10 mL of PBS. Solvent-mediated: EDOT was dissolved in the organic solvent first, and DA was dissolved in PBS. The two solutions were then combined. The solution was purged with nitrogen for 10 min prior to electropolymerization. Potentiostatic deposition was run at 1 V until reaching 50 mC total charge deposited. For PEDOT:PSS, potentiostatic deposition at 1 V was performed until reaching 200 mC cm^−2^ charge density. The PEDOT:PSS molar ratio was 1:2.5, corresponding to 10 mM EDOT (10.7 µL) and 2.5 mM PSS (50 mg) in 10 mL deionized water. The edge irregularity observed in some electrodes is due to the thermal damage to the Kapton tape induced by laser processing. This leads to the rugged contours of the PEDOT:PDA electropolymerization areas, which is eliminated when integrating the coating into a standard lithography process as shown in Fig. 8.

### Electrochemical characterization

CV and EIS measurements were conducted with the CHI660E electrochemical working station and a three-electrode setup. CV measurements were taken in both 0.1 M KCl containing 2 mM [Fe(CN)_6_]^3−/4−^ and PBS, with a voltage sweep of -0.6 V to 0.8 V at a 100 mV s^−1^ scan rate and 1 mV sample interval. Five successive scans were performed to stabilize the voltammogram before each acquired measurement.

EIS measurements were performed in PBS by applying a sinusoidal voltage amplitude of 10 mV and no direct current potential (0 V relative to open circuit potential), over a frequency range of 1 Hz – 100 kHz. EIS spectra were fitted according to the constitutional equations of the components in the equivalent circuit model of Fig. 1E using Zahner software.

*CSC calculation:* cathodic CSC was calculated using CV data in [Fe(CN)_6_]^3−/4−^ for the negative current range:

3 $$CSC= {\int }_{{V}_{min}}^{{V}_{max}}i\left(V\right)dV$$

*EASA calculation*: EASA values were calculated using the Randles-Ševčík equation on the [Fe(CN)_6_]^3−/4−^ CV data as

4 $${i}_{p}=0.4463 nFAC\sqrt{\frac{nF\vartheta D}{RT}}$$

where i_p_ is the maximum observed peak current in the CV scan, n is the number of transferred electrons in the redox reaction (n = 1), A is the electrochemically active surface area in cm^2^, D is the diffusion coefficient of the [Fe(CN)_6_]^3−/4−^ redox probe in KCl solution (equal to 7.6 × 10^−^⁶ cm^2^ s^−1^, ^21^), C is the concentration of the [Fe(CN)_6_]^3−/4−^ in mol.cm^-3^ (2.10^–6^ mol cm^-3^), ϑ is the scan rate in V s^−1^ set to 0.1 V s^−1^, F is the Faraday constant (9.65 C mol^−1^), R is the gas constant (8.31 J K^−1^ mol^−1^), and T is the temperature in K. At 25 °C, the equation can be simplified to:

5 $$i_{p} = \left( {2.69 \times 10^{5} } \right)n^{\frac{3}{2}} A\sqrt D C\sqrt \vartheta \Leftrightarrow A = \frac{{i_{p} }}{{\left( {2.69 \times 10^{5} } \right)n^{\frac{3}{2}} \sqrt D C\sqrt \vartheta }}$$

### Voltage transients

The same three-electrode set up described previously in PBS was used in conjunction with a custom-built pulse generator connected to a power supply (Keysight E36234A). The electrode polarization was recorded using a digital oscilloscope (Keysight DSOX1204G). The data were acquired with Matlab. Current-controlled, biphasic pulses were applied between the working and the counter electrode (0.3 ms per phase pulsewidth, 1 s inter-pulse period, 0.06 ms inter-phase delay), while measuring the polarization across the working and reference electrodes. The interface polarization was estimated as the potential difference between the inter-phase singularity point and the inter-pulse voltage.

### Adhesion tests

Coating adhesion was evaluated by immersing the samples in deionized water and exposed to an ultrasonic bath (Fisher Scientific FB15051) at 35 °C, with 37 kHz frequency and 320 W peak power.

### Topography characterization

Surface morphology was observed by scanning electron microscopy (SEM Hitachi SU 8230) at 5 kV. The topography and the roughness of the coating was investigated by atomic force microscopy (AFM XE-200, Park Systems) on a 25 µm^2^ scan area. The roughness values were determined using XEI software, which accounted for all surface variations, including both the dips and peaks, across the entire scan area. The thickness was determined using a Bruker Dektak profilometer. The insulating Kapton tape was carefully removed prior to thickness measurements. Each sample was scanned three times: on the top, middle and bottom of the coated area. The samples were then rotated 90° and the measurement was repeated as previously described.

### Error calculations

Unless otherwise stated, all data values are expressed as mean ± standard error. The standard deviation was determined from the various measured values on identical samples as well as across several samples. The standard error was then calculated by dividing the standard deviation by the square root of number of measured values for this sample.

## Supplementary Information

Supplementary Information.

## Data Availability

The datasets generated during the current study are available in the Zenodo repository, 10.5281/zenodo.17127500.

## References

[1] Iqbal, S. & Ahmad, S. Recent development in hybrid conducting polymers: Synthesis, applications and future prospects. *J. Ind. Eng. Chem.* **60**, 53–84 (2018).

[2] Guo, B. & Ma, P. X. Conducting Polymers for Tissue Engineering. *Biomacromol* **19**, 1764–1782 (2018). 10.1021/acs.biomac.8b00276 PMC6211800 29684268

[3] Groenendaal, L., Jonas, F., Freitag, D., Pielartzik, H. & Reynolds, J. R. Poly(3,4-ethylenedioxythiophene) and Its Derivatives: Past, Present, and Future. *Adv. Mater.* **12**, 481–494 (2000).

[4] Cui, X. & Martin, D. C. Electrochemical deposition and characterization of poly(3,4-ethylenedioxythiophene) on neural microelectrode arrays. *Sens. Actuators, B Chem.* **89**, 92–102 (2003).

[5] Rajesh, M. et al. Electrochemical polymerization of chloride doped PEDOT hierarchical porous nanostructure on graphite as a potential electrode for high performance supercapacitor. *Electrochim. Acta* **354**, 136669 (2020).

[6] Bodart, C. et al. Electropolymerized Poly(3,4-ethylenedioxythiophene) (PEDOT) Coatings for Implantable Deep-Brain-Stimulating Microelectrodes. *ACS Appl. Mater. Interfaces* **11**, 17226–17233 (2019). 30978001 10.1021/acsami.9b03088

[7] Vallejo-Giraldo, C. et al. Attenuated Glial Reactivity on Topographically Functionalized Poly(3,4-Ethylenedioxythiophene):P-Toluene Sulfonate (PEDOT:PTS) Neuroelectrodes Fabricated by Microimprint Lithography. *Small* **14**, 1800863 (2018). 10.1002/smll.201800863 29862640

[8] Asplund, M., von Holst, H. & Inganäs, O. Composite biomolecule/PEDOT materials for neural electrodes. *Biointerphases* **3**, 83–93 (2008). 20408704 10.1116/1.2998407

[9] Chakravarthy, S., Balasubramani, P. P., Mandali, A., Jahanshahi, M. & Moustafa, A. A. The many facets of dopamine: Toward an integrative theory of the role of dopamine in managing the body’s energy resources. *Physiol. Behav.* **195**, 128–141 (2018). 30031088 10.1016/j.physbeh.2018.06.032

[10] JuárezOlguín, H., CalderónGuzmán, D., Hernández García, E. & BarragánMejía, G. The Role of Dopamine and Its Dysfunction as a Consequence of Oxidative Stress. *Oxid Med Cell Longev* **2016**, 9730467 (2016). 26770661 10.1155/2016/9730467 PMC4684895

[11] Ryu, J. H., Messersmith, P. B. & Lee, H. Polydopamine Surface Chemistry: A Decade of Discovery. *ACS Appl. Mater. Interfaces* **10**, 7523–7540 (2018). 29465221 10.1021/acsami.7b19865 PMC6320233

[12] Lee, H., Rho, J. & Messersmith, P. B. Facile Conjugation of Biomolecules onto Surfaces via Mussel Adhesive Protein Inspired Coatings. *Adv. Mater.* **21**, 431–434 (2009). 19802352 10.1002/adma.200801222 PMC2755254

[13] Kim, S., Lee, S., Park, J. & Lee, J. Y. Electrochemical Co-deposition of Polydopamine/Hyaluronic Acid for Anti-biofouling Bioelectrodes. *Frontiers in Chemistry* **7**, (2019). 10.3389/fchem.2019.00262 PMC6503041 31114782

[14] Schiavone, G. et al. Guidelines to Study and Develop Soft Electrode Systems for Neural Stimulation. *Neuron* **108**, 238–258 (2020). 33120021 10.1016/j.neuron.2020.10.010

[15] Franks, W., Schenker, I., Schmutz, P. & Hierlemann, A. Impedance characterization and modeling of electrodes for biomedical applications. *IEEE Trans Biomed Eng* **52**, 1295–1302 (2005). 16041993 10.1109/TBME.2005.847523

[16] Abidian, M. R. & Martin, D. C. Experimental and theoretical characterization of implantable neural microelectrodes modified with conducting polymer nanotubes. *Biomaterials* **29**, 1273–1283 (2008). 18093644 10.1016/j.biomaterials.2007.11.022 PMC2692518

[17] Bard, A. J., Faulkner, L. R. & White, H. S. *Electrochemical Methods: Fundamentals and Applications*. (John Wiley & Sons, 2022).

[18] Schiavone, G., Vachicouras, N., Vyza, Y. & Lacour, S. P. Dimensional scaling of thin-film stimulation electrode systems in translational research. *J. Neural Eng.* **18**, 046054 (2021). 10.1088/1741-2552/abf607 33831857

[19] Kim, R. & Nam, Y. Polydopamine-doped conductive polymer microelectrodes for neural recording and stimulation. *J. Neurosci. Methods* **326**, 108369 (2019). 31326604 10.1016/j.jneumeth.2019.108369

[20] Castagnola, V. et al. Parylene-based flexible neural probes with PEDOT coated surface for brain stimulation and recording. *Biosens. Bioelectron.* **67**, 450–457 (2015). 25256782 10.1016/j.bios.2014.09.004

[21] Rezaei, B., Saghir, S., Pan, J. Y., Davidsen, R. S. & Keller, S. S. Selective Passivation of Three-Dimensional Carbon Microelectrodes by Polydopamine Electrodeposition and Local Laser Ablation. *Micromachines (Basel)* **13**, 371 (2022). 10.3390/mi13030371 PMC8950879 35334663

[22] Stöckle, B. et al. Precise Control of Polydopamine Film Formation by Electropolymerization. *Macromol. Symp.* **346**, 73–81 (2014).

[23] Yu, H. et al. Electrochemical Realization of 3D Interconnected MoS/PPy Nanowire Frameworks as Sulfur-Equivalent Cathode Materials for Li-S Batteries. *ENERGY & ENVIRONMENTAL MATERIALS* **7**, e12539 (2024).

[24] Becker, E. et al. All-organic thin-film transistors patterned by means of selective electropolymerization. *Appl. Phys. Lett.* **83**, 4044–4046 (2003).

[25] Niederhoffer, T., Vanhoestenberghe, A. & Lancashire, H. T. Methods of poly(3,4)-ethylenedioxithiophene (PEDOT) electrodeposition on metal electrodes for neural stimulation and recording. *J. Neural Eng.* **20**, 011002 (2023). 10.1088/1741-2552/acb084 36603213

[26] Teixeira, H. J., Dias, C., Veloso, R. C., Apolinário, A. & Ventura, J. Tuning PEDOT:PSS low-impedance thin films with high charge injection for microelectrodes applications. *Prog. Org. Coat.* **168**, 106894 (2022).

[27] Elschner, A., Kirchmeyer, S., Lovenich, W., Merker, U. & Reuter, K. *PEDOT: Principles and Applications of an Intrinsically Conductive Polymer*. (CRC Press, 2010).

[28] Puguan, J. M. C., Rathod, P. V., More, P. P. & Kim, H. Highly soluble electroactive ethylenedioxythiopene (EDOT)-based copolymer obtained via ‘click’ copolymerization. *Polymer* **226**, 123846 (2021).

[29] Wang, J. et al. Electropolymerization of dopamine for surface modification of complex-shaped cardiovascular stents. *Biomaterials* **35**, 7679–7689 (2014). 24929615 10.1016/j.biomaterials.2014.05.047

[30] Li, S. et al. Properties of Electropolymerized Dopamine and Its Analogues. *Langmuir* **35**, 1119–1125 (2019). 30137995 10.1021/acs.langmuir.8b01444

[31] Rozhkova, X. S., Aimukhanov, A. K., Ilyassov, B. R. & Zeinidenov, A. K. The role of alcoholic solvents in PEDOT:PSS modification as hole transport layers for polymer solar cells. *Opt. Mater.* **131**, 112708 (2022).

[32] You, I. et al. Polydopamine coating in organic solvent for material-independent immobilization of water-insoluble molecules and avoidance of substrate hydrolysis. *J. Ind. Eng. Chem.* **46**, 379–385 (2017).

[33] Jiang, X., Wang, Y. & Li, M. Selecting water-alcohol mixed solvent for synthesis of polydopamine nano-spheres using solubility parameter. *Sci Rep* **4**, 6070 (2014). 25317902 10.1038/srep06070 PMC4677634

[34] Poverenov, E., Li, M., Bitler, A. & Bendikov, M. Major Effect of Electropolymerization Solvent on Morphology and Electrochromic Properties of PEDOT Films. *Chem. Mater.* **22**, 4019–4025 (2010).

[35] Dimitriev, O. P., Grinko, D. A., Noskov, Yu. V., Ogurtsov, N. A. & Pud, A. A. PEDOT:PSS films—Effect of organic solvent additives and annealing on the film conductivity. *Synth. Met.* **159**, 2237–2239 (2009).

[36] Zhang, Y., Li, L. & He, B. Influences of solvents and monomer concentrations on the electrochemical performance and structural properties of electrodeposited PEDOT films: a comparative study in water and acetonitrile. *RSC Adv.* **14**, 30045–30054 (2024). 39309656 10.1039/d4ra03543g PMC11413736

[37] Cogan, S. F. Neural Stimulation and Recording Electrodes. *Annu. Rev. Biomed. Eng.* **10**, 275–309 (2008). 18429704 10.1146/annurev.bioeng.10.061807.160518

[38] Shuja, A. et al. Supercapacitors for energy storage applications: Materials, devices and future directions: A comprehensive review. *J. Alloy. Compd.* **1009**, 176924 (2024).

[39] (nee Włodarczyk), K. C., Karczewski, J. & Jasiński, P. Influence of electropolymerization conditions on the morphological and electrical properties of PEDOT film. *Electrochimica Acta* **176**, 156–161 (2015).

[40] Boehler, C., Oberueber, F., Schlabach, S., Stieglitz, T. & Asplund, M. Long-Term Stable Adhesion for Conducting Polymers in Biomedical Applications: IrOx and Nanostructured Platinum Solve the Chronic Challenge. *ACS Appl. Mater. Interfaces* **9**, 189–197 (2017). 27936546 10.1021/acsami.6b13468

[41] Tian, F. et al. Design of adhesive conducting PEDOT-MeOH:PSS/PDA neural interface via electropolymerization for ultrasmall implantable neural microelectrodes. *J. Colloid Interface Sci.* **638**, 339–348 (2023). 36746052 10.1016/j.jcis.2023.01.146

[42] Aqrawe, Z., Montgomery, J., Travas-Sejdic, J. & Svirskis, D. Conducting polymers for neuronal microelectrode array recording and stimulation. *Sens. Actuators, B Chem.* **257**, 753–765 (2018).

[43] Schiavone, G. et al. Soft, Implantable Bioelectronic Interfaces for Translational Research. *Adv. Mater.* **32**, 1906512 (2020). 10.1002/adma.201906512 32173913

[44] Schiavone, G. *et al.* Long-term functionality of a soft electrode array for epidural spinal cord stimulation in a minipig model. in *2018 40th Annual International Conference of the IEEE Engineering in Medicine and Biology Society (EMBC)* 1432–1435 (2018). 10.1109/EMBC.2018.8512584. 10.1109/EMBC.2018.8512584 30440661

[45] Ji, B. et al. Flexible bioelectrodes with enhanced wrinkle microstructures for reliable electrochemical modification and neuromodulation in vivo. *Biosens. Bioelectron.* **135**, 181–191 (2019). 31022595 10.1016/j.bios.2019.04.025

[46] Lim, C. et al. Nanomechanics of Poly(catecholamine) Coatings in Aqueous Solutions. *Angew. Chem. Int. Ed.* **55**, 3342–3346 (2016). 10.1002/anie.201510319 26833974

[47] Tiu, B. D. B., Delparastan, P., Ney, M. R., Gerst, M. & Messersmith, P. B. Cooperativity of Catechols and Amines in High-Performance Dry/Wet Adhesives. *Angew. Chem.* **132**, 16759–16767 (2020). 10.1002/anie.202005946 32537907

[48] Lyu, Q., Hsueh, N. & Chai, C. L. L. The Chemistry of Bioinspired Catechol(amine)-Based Coatings. *ACS Biomater. Sci. Eng.* **5**, 2708–2724 (2019). 33405603 10.1021/acsbiomaterials.9b00281

[49] Saiz-Poseu, J., Mancebo-Aracil, J., Nador, F., Busqué, F. & Ruiz-Molina, D. The Chemistry behind Catechol-Based Adhesion. *Angew. Chem. Int. Ed.* **58**, 696–714 (2019). 10.1002/anie.201801063 29573319

[50] Barclay, T. G., Hegab, H. M., Clarke, S. R. & Ginic-Markovic, M. Versatile Surface Modification Using Polydopamine and Related Polycatecholamines: Chemistry, Structure, and Applications. *Adv. Mater. Interfaces* **4**, 1601192 (2017).

[51] Kim, S. et al. Electrically Conductive Polydopamine-Polypyrrole as High Performance Biomaterials for Cell Stimulation in Vitro and Electrical Signal Recording in Vivo. *ACS Appl. Mater. Interfaces* **10**, 33032–33042 (2018). 30192136 10.1021/acsami.8b11546

[52] Carter, J. L., Kelly, C. A. & Jenkins, M. J. Enhanced adhesion of PEDOT:PSS to substrates using polydopamine as a primer. *Polym J* **56**, 115–120 (2024).

[53] Kim, S., Jang, L. K., Park, H. S. & Lee, J. Y. Electrochemical deposition of conductive and adhesive polypyrrole-dopamine films. *Sci Rep* **6**, 30475 (2016). 27459901 10.1038/srep30475 PMC4962031

[54] Peckerar, M., Shamma, S. A., Rebbert, M., Kosakowski, J. & Isaacson, P. Passive microelectrode arrays for recording of neural signals: A simplified fabrication process. *Rev. Sci. Instrum.* **62**, 2276–2280 (1991).

[55] Stieglitz, T., Beutel, H., Keller, R., Schuettler, M. & Meyer, J.-U. Flexible, polyimide-based neural interfaces. in *Proceedings of the Seventh International Conference on Microelectronics for Neural, Fuzzy and Bio-Inspired Systems* 112–119 (1999). 10.1109/MN.1999.758853.

[56] Owens, A. L. et al. Multi-electrode array for measuring evoked potentials from surface of ferret primary auditory cortex. *J. Neurosci. Methods* **58**, 209–220 (1995). 7475229 10.1016/0165-0270(94)00178-j

[57] King, Z. A., Shaw, C. M., Spanninga, S. A. & Martin, D. C. Structural, chemical and electrochemical characterization of poly(3,4-Ethylenedioxythiophene) (PEDOT) prepared with various counter-ions and heat treatments. *Polymer* **52**, 1302–1308 (2011). 21394224 10.1016/j.polymer.2011.01.042 PMC3049552

[58] Wilks, S. J., Richardson-Burn, S. M., Hendricks, J. L., Martin, D. & Otto, K. J. Poly(3,4-ethylene dioxythiophene) (PEDOT) as a micro-neural interface material for electrostimulation. *Front. Neuroeng.* **2**, (2009). 10.3389/neuro.16.007.2009 PMC2697029 19543541

[59] Sun, T. et al. Flexible IrOx neural electrode for mouse vagus nerve stimulation. *Acta Biomater.* **159**, 394–409 (2023). 36669547 10.1016/j.actbio.2023.01.026 PMC10823593

[60] Leber, M. et al. Long term performance of porous platinum coated neural electrodes. *Biomed Microdevices* **19**, 62 (2017). 28688070 10.1007/s10544-017-0201-4

[61] Rocha, J. F., Hasimoto, L. H. & Santhiago, M. Recent progress and future perspectives of polydopamine nanofilms toward functional electrochemical sensors. *Anal Bioanal Chem* 1–18 (2023) 10.1007/s00216-023-04522-z. 10.1007/s00216-023-04522-z PMC9841946 36645457

[62] Szewczyk, J., Aguilar-Ferrer, D. & Coy, E. Polydopamine films: Electrochemical growth and sensing applications. *Eur. Polymer J.* **174**, 111346 (2022).

[63] Huang, J. et al. Improving the efficiency and stability of inverted perovskite solar cells with dopamine-copolymerized PEDOT:PSS as a hole extraction layer. *J. Mater. Chem. A* **5**, 13817–13822 (2017).

[64] Salgado, R., del Rio, R., del Valle, M. A. & Armijo, F. Selective electrochemical determination of dopamine, using a poly(3,4-ethylenedioxythiophene)/polydopamine hybrid film modified electrode. *J. Electroanal. Chem.* **704**, 130–136 (2013).

[65] Salgado, R., del Valle, M. A., Duran, B. G., Pardo, M. A. & Armijo, F. Optimization of dopamine determination based on nanowires PEDOT/polydopamine hybrid film modified electrode. *J Appl Electrochem* **44**, 1289–1294 (2014).

[66] Islam, G. M. N., Ali, M. A. & Collie, S. Polydopamine Treated and PEDOT:PSS Coated Wash Durable Conductive Textiles for Wearable Applications. *Fibers Polym* **23**, 914–924 (2022).

[67] Elanthamilan, E. et al. Fabrication of polydopamine/polyaniline decorated multiwalled carbon nanotube composite as multifunctional electrode material for supercapacitor applications. *Synth. Met.* **298**, 117423 (2023).

[68] Deng, Z. et al. Biofunction of Polydopamine Coating in Stem Cell Culture. *ACS Appl. Mater. Interfaces* **13**, 10748–10759 (2021). 33594879 10.1021/acsami.0c22565

