# Supplementary material for: Electropolymerised PEDOT:Polydopamine enables high-performance bioelectrode coatings
Source: Sci Rep. 2025 Oct 8;15:35130. doi: 10.1038/s41598-025-19124-1 (PMC12508230; doi:10.1038/s41598-025-19124-1)
Supplement: Supplementary file 1 — Supplementary Information. [file 41598_2025_19124_MOESM1_ESM.pdf]

Supplementary Information

Electropolymerised PEDOT:Polydopamine enables High-Performance Bioelectrode Coatings

Saloua Saghir, [1] Jairo Ramirez-Sarabia, [1] Kristin Imenes, [1] Giuseppe Schiavone[1]*
[1] Department of Microsystems, Faculty of Technology, Natural Sciences and Maritime Sciences, University of South-Eastern Norway, Horten, Norway

* Corresponding author: giuseppe.schiavone@usn.no

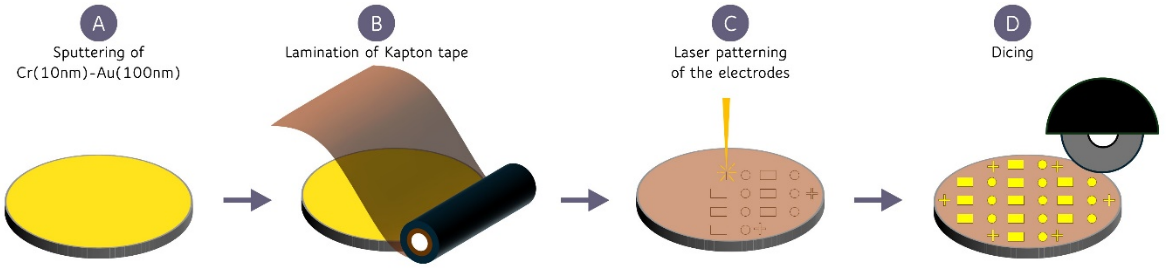

Fig. S1. Process flow for the fabrication of test electrodes. (A) Sputtering of a Cr-Au 10-100 nm adhesion-conductor thin film layer on a thermally oxidized 4" Si wafer. **(B)** Lamination of a 65 µm Kapton tape insulation over the entire wafer area. **(C)** CO_2 laser patterning of the Kapton insulation to define electrode vias and pads for electrical connection. (**D**) Wafer dicing to singulate individual dies (1 x 2.5 cm^2) with one test electrode on each.

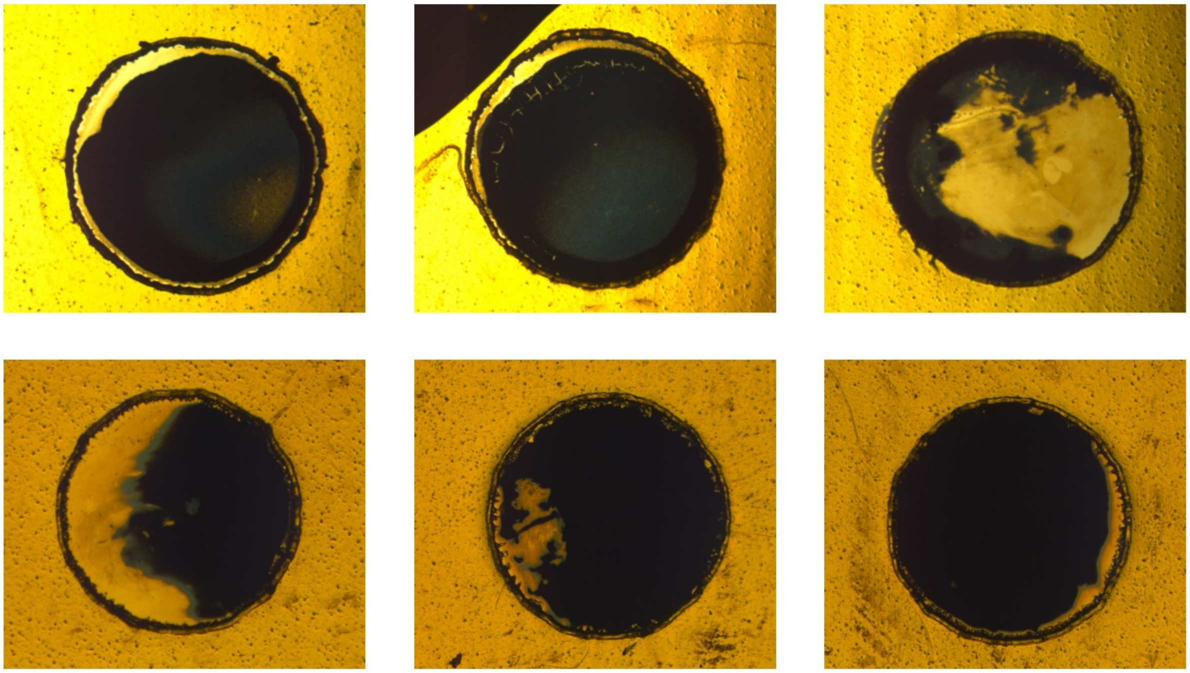

Fig. S2. Representative optical micrographs of nominally identical PEDOT:PDA electrodes (2 mm diameter) electropolymerized in PBS electrolyte.

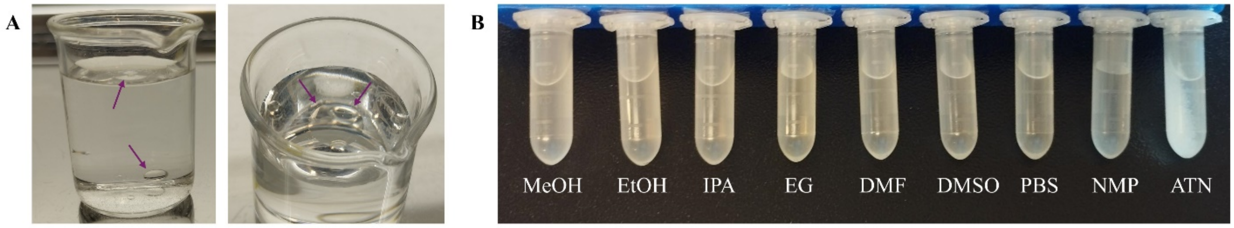

Fig. S3. Solubility of EDOT and DA in different solvents. (A) Photographs of EDOT monomer dissolution in a 10 ml beaker filled with PBS. Arrows indicate EDOT monomer solution. **(B)** Photograph of DA dissolution in 1.5 ml Eppendorf vials containing various solvents.

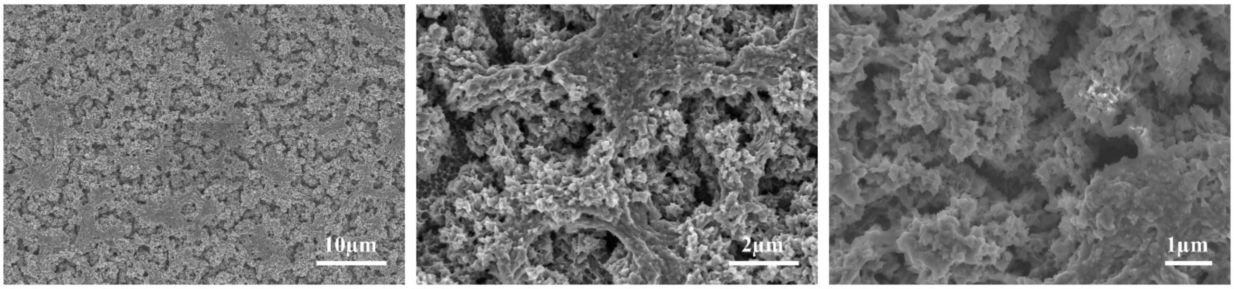

Fig. S4. SEM images at different magnifications of PEDOT:PDA electrodes electropolymerized in 90:10 %v:v PBS:ATN electrolyte. No qualitative difference in structure is observed compared to the samples deposited in electrolytes containing IPA, MeOH, EtOH or EG solvent.

Table S1. Approximate pH values of the solvents in presence of DA monomers. Values measured by pH indicator paper.

Solvent	PBS	ATN	DMF	DMSO	EG	EtOH	IPA	MeOH	NMP
pH	7	5	6	7	5	4	4	4	5

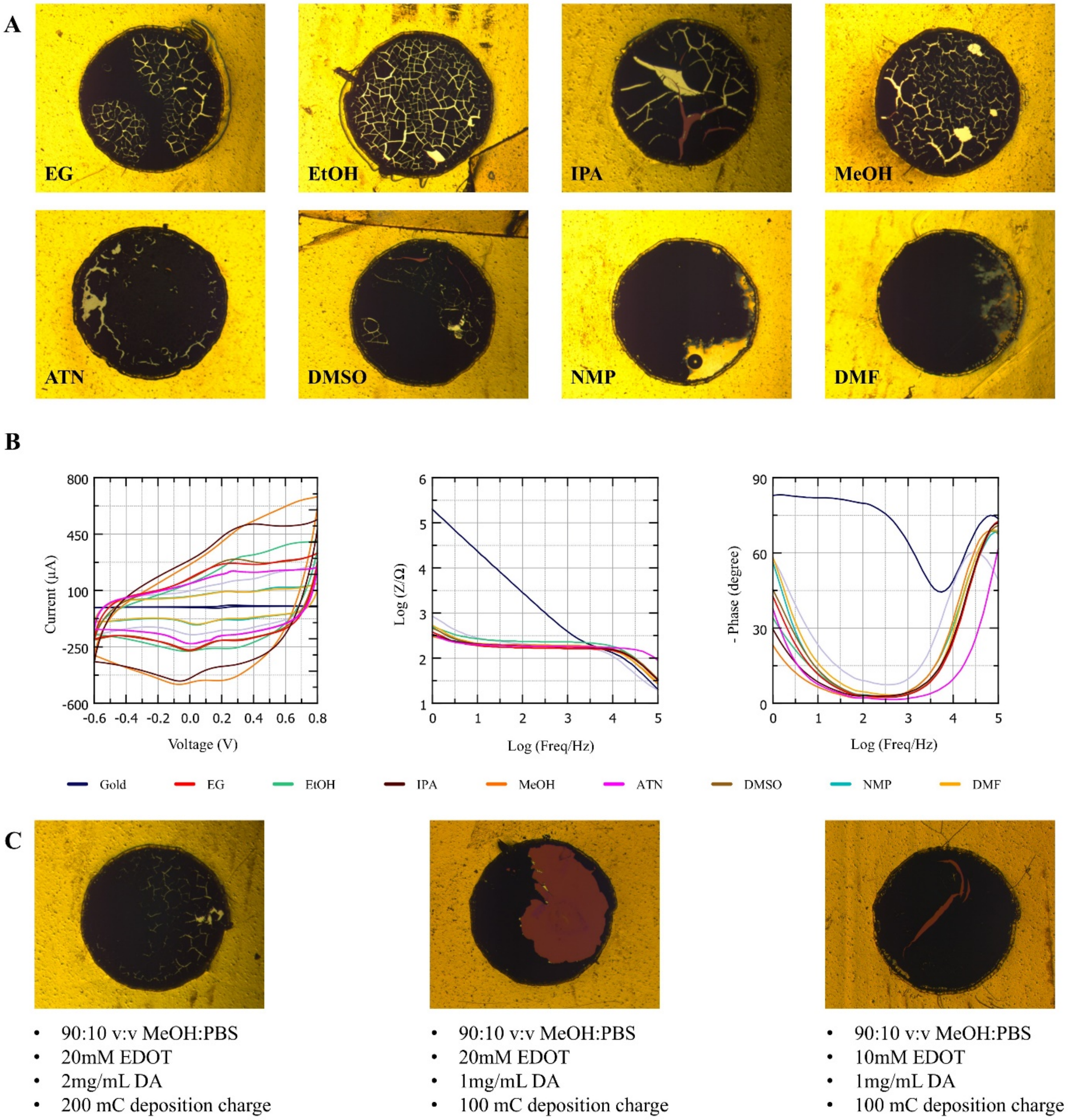

Fig. S5. Optical and electrochemical characterization of 2 mm diameter electrodes for different monomer concentrations. (**A**) Optical micrographs of the coated electrodes in various solvents using monomer concentrations of 20 mM EDOT and 2 mg mL^{-1} DA. (**B**) Electrochemical characterization of the above coated electrodes (CV – left, EIS modulus – centre, EIS phase – right). (**C**) Optical micrographs of electrodes coated at different monomer concentrations and deposition charge.

Table S2. Mann–Whitney U test p-values comparing CSC values of the PEDOT:PDA coatings prepared with different solvents. In red the values p<0.05.

	ATN	DMF	DMSO	EG	EtOH	MeOH	IPA	NMP
**ATN**								
**DMF**	0.029							
**DMSO**	0.057	0.029						
**EG**	0.2	0.029	0.029					
**EtOH**	0.343	0.029	0.057	0.89				
**MeOH**	0.886	0.029	0.11	0.69	0.74			
**IPA**	0.486	0.029	0.029	0.2	0.89	0.69		
**NMP**	0.029	0.029	0.029	0.029	0.029	0.029	0.029	

Table S3. Mann–Whitney U test p-values comparing impedance modulus values of the PEDOT:PDA coatings prepared with different solvents. In red the values p<0.05

	ATN	DMF	DMSO	EG	EtOH	MeOH	IPA	NMP
**ATN**								
**DMF**	0.029							
**DMSO**	0.34	0.029						
**EG**	1	0.029	0.029					
**EtOH**	0.89	0.029	0.057	0.69				
**MeOH**	1	0.029	0.2	1	0.89			
**IPA**	1	0.029	0.11	1	0.69	0.89		
**NMP**	0.11	0.029	0.057	0.029	0.029	0.029	0.029	

Table S4. Mann–Whitney U test p-values comparing EASA values of the PEDOT:PDA coatings prepared with different solvents. In red the values p<0.05

	ATN	DMF	DMSO	EG	EtOH	MeOH	IPA	NMP
ATN								
DMF	0.029							
DMSO	0.057	0.029						
EG	0.11	0.029	0.029					
EtOH	0.34	0.029	0.057	0.69				
MeOH	0.8	0.029	0.11	0.31	1			
IPA	0.34	0.029	0.029	0.49	1	0.63		
NMP	0.029	0.029	0.057	0.029	0.029	0.029	0.029	

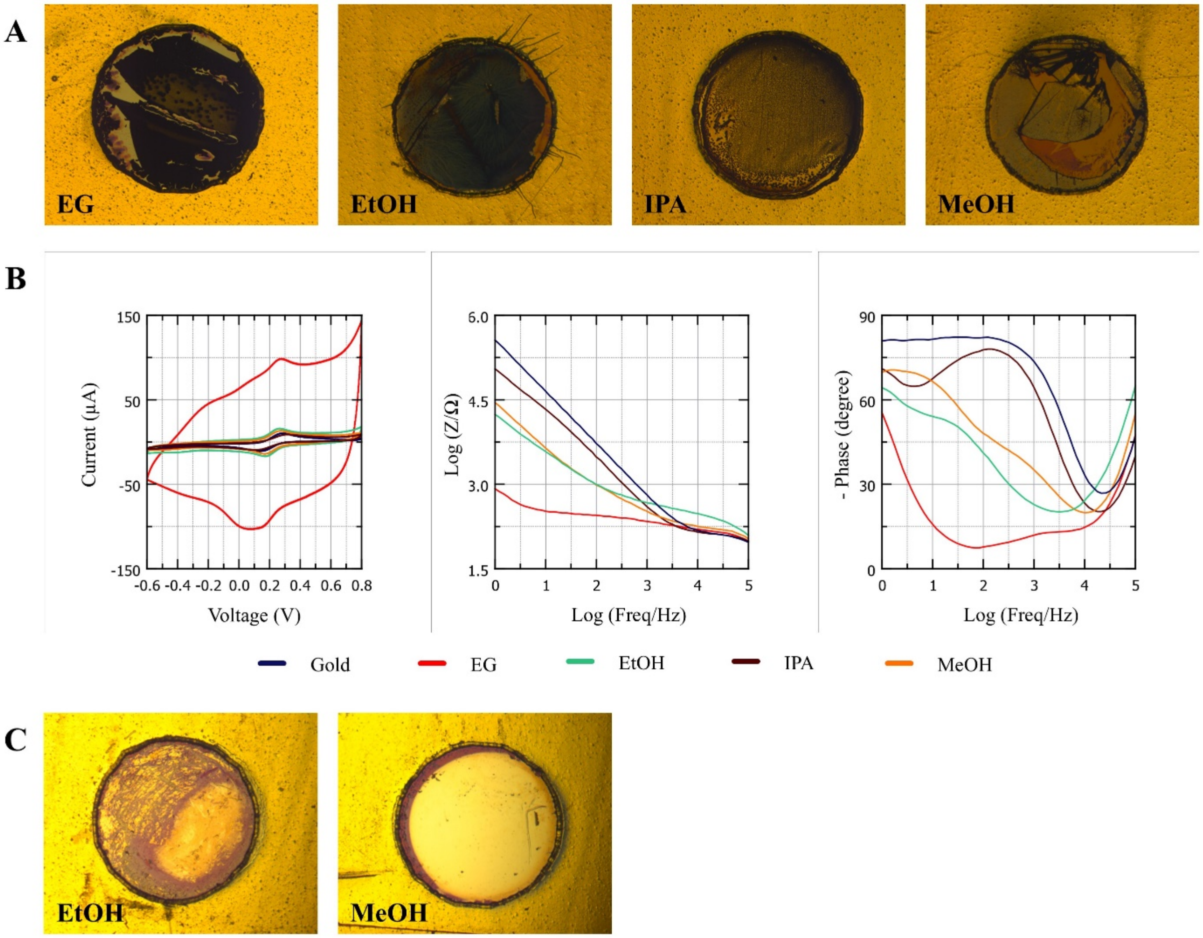

Fig. S6. Optical and electrochemical characterization of 2 mm diameter electrodes for different electrolyte composition. (**A**) Optical micrographs of the electrodes coated in 50:50 %v:v PBS: Organic solvents. (**B**) Electrochemical characterization of the above coated electrodes (CV – left, EIS modulus – centre, EIS phase – right). (**C**) Optical micrographs of electrodes coated in pure EtOH and MeOH.

Table S5. Mann–Whitney U test p-values comparing the thickness of the PEDOT:PDA coatings prepared with the fours best different solvents.

	**EtOH**	**IPA**	**EG**	**MeOH**
**EtOH**				
**IPA**	0.1			
**EG**	0.4	0.1		
**MeOH**	0.1	0.1	0.1	

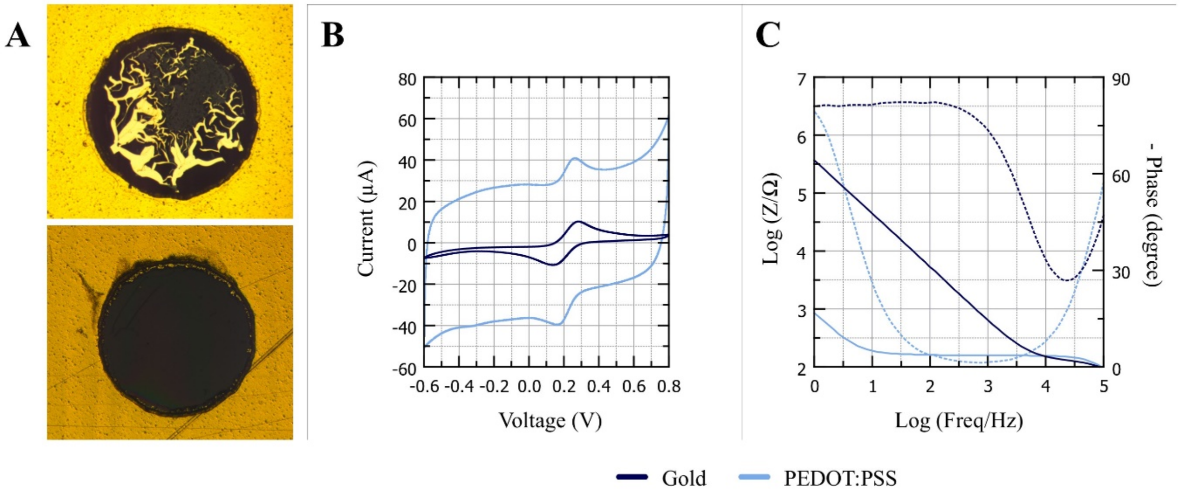

Fig. S7. Optical and electrochemical characterization of PEDOT:PSS control electrodes. (**A**) Optical micrographs of 2 mm diameter PEDOT:PSS control electrodes. Top: electrode coated with 1592 mC cm^{-2} potentiostatic deposition charge, leading to the cracks and inhomogeneities. Bottom: electrode coated at 200 mC cm^{-2} potentiostatic deposition charge, leading to a homogeneous, uniform coating. (**B**) CV scans of the bare Au and PEDOT:PSS coated electrodes at 100 mV s^{-1} scan rate. (**C**) Impedance modulus (solid lines, left y axis) and phase (dashed lines, right y axis) for bare Au and PEDOT:PSS coated electrodes.

Table S6. EIS fitting parameters and extracted C_{eff} value at 1 Hz. Effective capacitance C_{eff} at 1 Hz was calculated using the best fit Q and n values (see Methods for error calculation).

**Equivalent circuit parameters**	**R_s [Ω]**	**Q [S s^n]**	**n**	**C_p [F]**	**C_{eff} at 1 Hz [F]**
**Gold**	126.2 ± 1.3	8.9E-07 ± 9.4E-9	0.919 ± 0.002	1.20E-08 ± 3.26E-10	7.67E-07
**PEDOT:PDA (EG)**	157.5 ± 1.4	4.2E-04 ± 1.9E-5	0.916 ± 0.014	7.38E-09 ± 4.39E-10	3.60E-04

Table S7. C_{eff} at 1 Hz and cathodic CSC values of EG-electropolymerized PEDOT:PDA electrodes.

**Ceff (mF) at 1 Hz**	**CSC (mC cm^{-2})**
0.550	30.08
0.477	33.37
0.483	33.64
0.578	35.33
0.672	38.74
0.723	39.4
0.362	40.22
0.589	41.2
0.536	41.43
0.308	41.46
0.603	41.93
0.544	42.73
0.731	43.41
0.579	43.52
0.669	44.51
0.604	45.08
0.536	45.44
0.523	45.62
0.489	47.14
0.589	48.02
0.579	49.96
0.589	51.79

Immersion test

One PEDOT:PDA sample was immersed in PBS heated at 57°C, corresponding to an acceleration factor of 4 according to the 10-degree rule for Arrhenius processes [1]. After immersion for 3 weeks, the electrode was electrochemically characterized. The electrode retained their characteristic electrochemical signature, albeit with a decrease in current response (amounting to a 28% CSC decrease) and a 45% increase in impedance at 1 Hz after immersion.

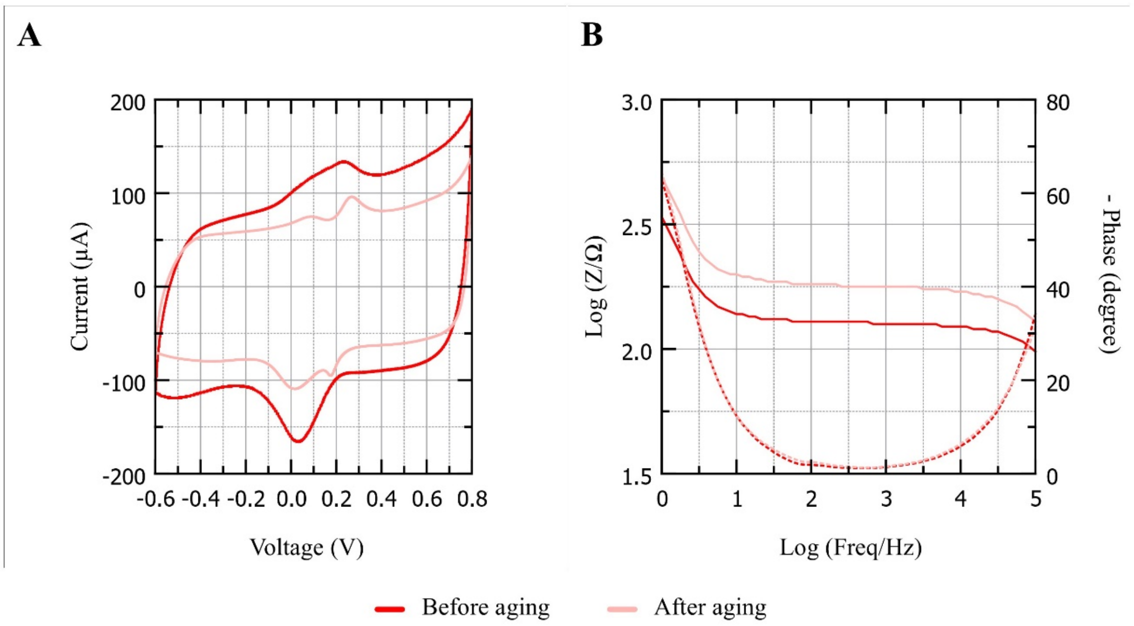

Fig. S8. 3-week immersion test in PBS at 57°C. (A) CV scans at 100 mV s^{-1} scan rate; **(B)** impedance modulus (solid lines, left y axis) and phase (dashed lines, right y axis) before and after immersion.

Table S8. Optimized electrolyte composition and deposition parameters for PEDOT:PDA electropolymerization.

Monomer concentrations	Electrolyte composition	Deposition charge
DA: 1 mg mL^{-1}<br>EDOT: 10 mM	90:10 %v:v<br>PBS:Ethylene Glycol	50 mC (1592 mC cm^{-2})

Table S9. Summary of PBS to organic solvent volumetric ratios tested for different electrolyte solvents for PEDOT:PDA electropolymerization.

Organic solvents	PBS: Organic solvent volumetric ratio	
**ATN**		90:10
**DMF**		90:10
**DMSO**		90:10
**NMP**		90:10
**EtOH**	50:50	90:10
**EG**	50:50	90:10
**IPA**	50:50	90:10
**MeOH**	50:50	90:10

References:

1. Muhamad, I. I. *et al.* 8 - Accelerated testing methodology for long-term life prediction of cellulose-based polymeric composite materials. in *Durability and Life Prediction in Biocomposites, Fibre-Reinforced Composites and Hybrid Composites* (eds. Jawaid, M., Thariq, M. & Saba, N.) 149–171 (Woodhead Publishing, 2019). doi:10.1016/B978-0-08-102290-0.00008-8.
